# Improving disease management of patients with inflammatory bowel disease: the potential role of self-concordant health goals

**DOI:** 10.3389/fpsyg.2023.1115160

**Published:** 2023-07-03

**Authors:** Barbara Horvát, Anett Dávid, Viola Sallay, Beatrix Rafael, Sanela Njers, Kata Orbán, Tamás Molnár, Márta Csabai, Tamás Martos

**Affiliations:** ^1^Doctoral School of Clinical Medicine, University of Szeged, Szeged, Hungary; ^2^Department of Medicine, University of Szeged, Szeged, Hungary; ^3^Institute of Psychology, University of Szeged, Szeged, Hungary; ^4^Department of Preventive Medicine, University of Szeged, Szeged, Hungary; ^5^Department of Cognitive and Neuropsychology, University of Szeged, Szeged, Hungary; ^6^Department of Clinical Psychology, Károli Gáspár University of the Reformed Church, Budapest, Hungary

**Keywords:** health-related goals, self-concordance, health behavior, chronic disease, inflammatory bowel disease, trait anxiety

## Abstract

Inflammatory bowel diseases (IBD) are chronic gastrointestinal conditions that significantly impact patients’ quality of life. Previous research indicates that patients with IBD have a higher prevalence of anxiety compared to the general population and other chronic diseases. This pilot study aimed to investigate the relationships between goal integration, positive and negative emotions, goal self-efficacy, and trait anxiety as the outcome variable, focusing on patients’ self-management strategies. Drawing from the Self-Concordance Model (SCM) of Self-Determination Theory (SDT), the study explored how goal integration is associated with more fulfilling and enjoyable experiences and fewer negative emotions, ultimately improving psychological well-being. Health-related goals were evaluated using the Personal Project Analysis technique, while the State–Trait Anxiety Inventory was utilized to measure general anxiety levels. Among the 141 participants with inflammatory bowel disease, 96 reported having health-related goals. Of these, 66 were female (68.75%), and 30 were male participants (31.25%). Path analysis revealed a moderate negative association between self-concordance (SC) and negative emotions, which, in turn, predicted higher levels of trait anxiety. Furthermore, the alternative model tested indicated that trait anxiety predicted a lower level of self-concordance. Setting well-integrated health goals involves an internal capacity, enabling patients to experience less negative emotions during self-management activities. Anxiety can hinder individuals from accessing their inner needs, resulting in less self-concordant aspirations and more negative emotions. These findings may contribute to developing prevention and intervention programs to enhance IBD patients’ adherence to lifestyle changes, ultimately improving their overall well-being.

## 1. Introduction

The global prevalence of inflammatory bowel diseases (IBDs) is rising, with these chronic conditions affecting multiple organs and primarily targeting the intestinal tissues (Podolsky, 2002; Sartor, 2006; Park et al., 2019). IBD patients often experience abdominal pain, bloody diarrhea, fatigue, and frequent bowel movements, significantly impacting their daily lives (Dibley and Norton, 2013; Devlen et al., 2014). Therapy aims to alleviate symptoms, achieve remission, and improve the overall quality of life for patients (Habibi et al., 2017). Given the physical burden of the disease and the heightened psychological vulnerability, empowering patients with self-management strategies, including emotion regulation and disease management skills, is crucial. This paper presents the findings of a cross-sectional pilot study conducted among inflammatory bowel disease patients. Our main objective was to explore the role of striving for health goals in disease management by examining the associations between three key elements of the health goal-striving process (goal-related self-concordance, goal self-efficacy, and positive and negative emotions) and their relationship with general anxiety.

### 1.1. Psychological aspects of living with IBD

The symptoms of IBD and its associated medications profoundly disrupt patients’ daily activities and psychological well-being, affecting various aspects of their lives, such as work, school, family, relationships, and overall psychological health (Dibley and Norton, 2013; Devlen et al., 2014). Numerous studies have demonstrated that the prevalence of anxiety and depressive disorders is higher among IBD patients compared to the general population and other chronic diseases (Robertson et al., 1989; Addolorato et al., 1997; Katon and Ciechanowski, 2002; Katon et al., 2007; Kovács and Kovács, 2007; Scott et al., 2007; Graff et al., 2009; Byrne et al., 2017; Bhamre et al., 2018). These symptoms tend to worsen during disease relapses, and long-term anxiety levels have been associated with poorer IBD-related outcomes (Nahon et al., 2012; Selinger and Bannaga, 2015; Narula et al., 2019). Moreover, managing IBD requires specific self-management skills, including adhering to complex medication regimens, regular medical check-ups, cancer screenings, addressing medication side effects and extraintestinal symptoms, and making lifestyle adjustments (e.g., stress management, healthy eating, smoking cessation; von Wietersheim et al., 1992; Kane et al., 2001; Dudley-Brown, 2002). Effective disease management is crucial to minimize complications and prevent psychological distress (Dudley-Brown, 2002). While previous research has primarily focused on disease education interventions rather than self-management components (Barlow et al., 2010; Kemp, 2012), it is crucial to further investigate factors that can enhance self-management and improve patient adherence, especially considering the significant nonadherence rates and maladaptive coping strategies among IBD patients (Wagoner and Kavookjian, 2017).

### 1.2. Lifestyle change and health-related personal goals

Supporting lifestyle changes is a vital aspect of disease management for individuals with IBD. Various activities related to disease management, role adjustment, and emotional well-being can be framed as personal goals (Austin and Vancouver, 1996; Peterman and Lecci, 2007; Martos, 2009a). For IBD patients, these goals may include alleviating physical symptoms, maintaining disease remission, managing lifestyle changes (medication adherence, dietary modifications, smoking cessation, and regular physical activity), and improving mental health. Health goals serve as a tool for IBD patients to adapt to the necessary lifestyle changes imposed by the disease (Strecher et al., 1995; Mann et al., 2013). Although many studies have highlighted the impact of adopting a healthy lifestyle on the quality of life of IBD patients (Lo et al., 2021; Lamers et al., 2022; Schlee et al., 2022), the experiences of setting personal health goals have not yet been explored among this population.

### 1.3. Goal self-concordance

According to the Self-Determination Theory (SDT) of health behaviors (Ryan and Deci, 2000), intrinsic motivation is crucial in long-term adherence. SDT proposes a continuum of motivation, where autonomously regulated health goals are pursued out of intrinsic motives and are aligned with the individual’s integrated sense of self (Sheldon and Elliot, 1999; Judge et al., 2005). The Self-Concordance Model (SCM; Ryan et al., 1996) addresses the extent to which goals are integrated into the self, indicating a lower degree of control, higher autonomy, and consistency with one’s core values, talents, and needs (Sheldon and Elliot, 1998, 1999; Judge et al., 2005). According to SCM, higher self-integration leads to greater effort and improved goal implementation outcomes (Sheldon and Elliot, 1999). Individuals become more deeply engaged in their health management with more integrated goals, leading to more effective goal achievement (Vansteenkiste et al., 2005). Developing self-concordance regarding health goals can support lifestyle changes for people with IBD (Reed-Knight et al., 2011).

### 1.4. Goal self-efficacy

Self-efficacy (SE) is key to health behavior change (McAuley, 1993; Lorig and Holman, 2003; Fernández et al., 2009). SE refers to an individual’s belief in their ability to perform the behaviors required to manage a situation (Bandura, 1977, 2001). This motivational factor has been shown to influence goal progress and long-term commitment (Bandura, 1977; Sheldon and Elliot, 1998; Koestner et al., 2008). Previous research among IBD patients has demonstrated that SE is a relevant component of disease management and coping (Graff et al., 2016). Moreover, SE has been found to have a positive association with self-esteem and health-related quality of life and a negative association with depression and anxiety (Izaguirre et al., 2017). IBD patients with high self-efficacy are more likely to visit gastroenterologists regularly and be open to psychological support (Keefer et al., 2011). Both self-efficacy and self-concordance are important determinants of successful lifestyle change. The question remains about how these factors interact and which is a stronger predictor of successful goal pursuit.

### 1.5. Positive and negative emotions

Self-concordant striving not only affects optimism about goal attainment but also influences emotions during the process (Sheldon and Elliot, 1999; Sheldon et al., 2004, 2022; Sheldon and Lyubomirsky, 2006; Wang, 2009; Gaudreau, 2012). Goals that align with inner values, talents, and needs have the potential to fulfill basic psychological needs, thereby contributing to enhanced psychological well-being (Sheldon and Elliot, 1999; Sheldon, 2002). In comparison, individuals with non-concordant goals tend to experience lower levels of happiness, even if they manage to accomplish those goals (Sheldon and Elliot, 1999). Furthermore, self-concordant goals are perceived as more attainable, facilitating more effective goal pursuit (Werner et al., 2016). Moreover, happiness-related exercises are more effective when self-concordant (Dickerhoof, 2007). Self-concordant future events are associated with more positive and intense emotions (Ernst et al., 2018).

### 1.6. The present study

**Figure 1:**
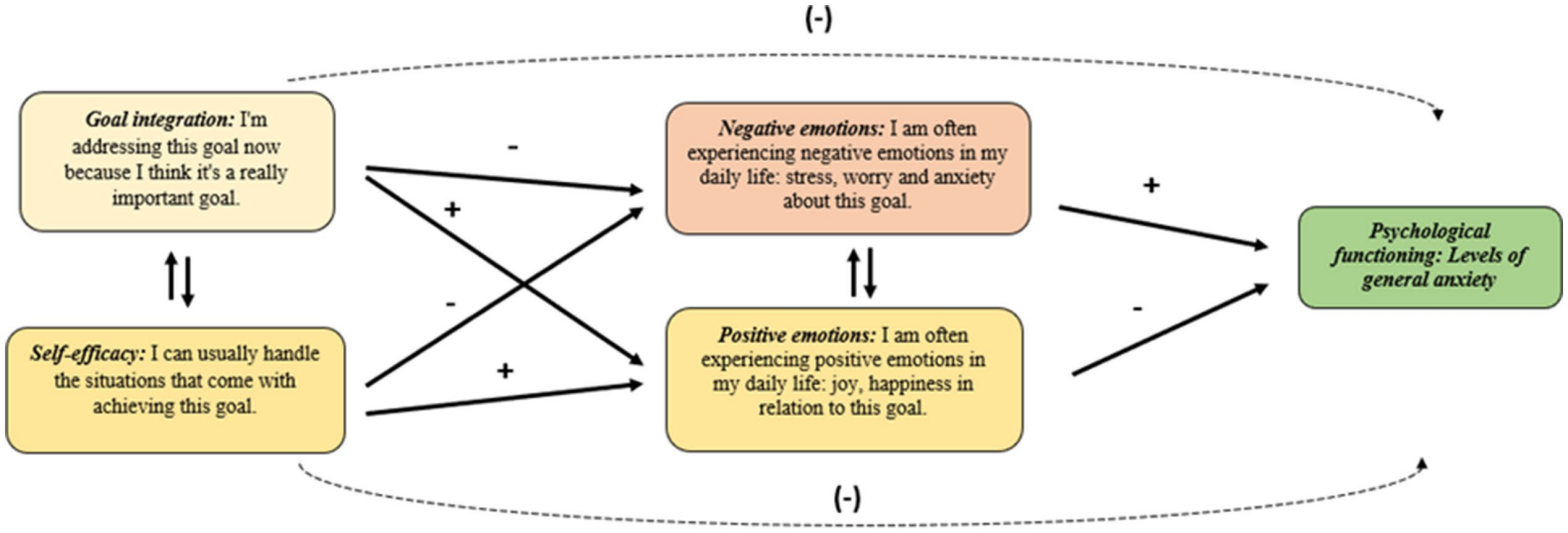
Goal integration’s role in emotional experiences of lifestyle change and better psychological functioning. “+” stands for a positive, and “−” for a reversed association.

This study proposes and examines a model (refer to Figure 1 for an overview and the hypothesized relationships between the variables). The level of self-integration of health goals reflects an individual’s internal capacity, as well-integrated health goals are closely aligned with their inner values, talents, and needs. Pursuing well-integrated goals allows individuals to engage in activities that genuinely reflect their motivations, resulting in more self-rewarding experiences filled with joy, pleasure, and a sense of flow while minimizing negative emotions such as frustration, sadness, or distress. Consequently, experiencing more positive and fewer negative emotions during the process of goal pursuit can act as a mediator in enhancing overall functioning. We assumed that self-concordance (SC) and goal self-efficacy (SE) would predict lower levels of anxiety by experiencing more positive and less negative emotions during goal implementation. We acknowledge the interdependence between self-concordance, goal self-efficacy, and positive and negative emotions. However, we do not propose a specific hypothesis regarding the direct impact of self-concordance or goal self-efficacy on trait anxiety. As this is a pilot study and causal relationships cannot be established, we propose an alternative model that may also be plausible. Drawing on previous research, we hypothesize that trait anxiety could be associated with more negative and less positive emotions, and also undermine both self-efficacy and self-concordance.

## 2. Methods

### 2.1. Participants

Participants were recruited from the Internal Medicine Department of the University of Szeged. Our target group was patients living with any type of inflammatory bowel disease. In sum, 141 IBD patients’ data were involved in the analysis. Data collection was carried out by filling in a paper-pencil questionnaire during patients’ regular check-ups in the hospital, due at a 6–8 weeks pace. Before receiving the questionnaire, the participants were informed about the participation conditions and requested to provide informed written consent.

### 2.2. Measures

#### 2.2.1. Personal project analysis

Patients were asked to list their health-related goals, select one of them and assess it according to the criteria provided. Sample personal health goals included domains such as sport (Example: *“I definitely need to start doing some form of exercise regularly.”*), weight management (Example: *“I want to lose weight.”*), eating habits (Example: *“Greater adherence to the diet”*), reduce smoking (Example: *“I want to quit smoking.”*), mental health (Example: *“Find a better work-life balance.”*), and sleeping (Example: *“Get 8 h of sleep every night.”*). The health goals were rated according to the following criteria (Little, 1993; Martos, 2009b).

##### 2.2.1.1. Goal self-concordance

Goal self-concordance refers to the extent to which the person has internalized the goal. It was calculated from the subtraction of two items of controlled motivation (External regulation: *“One of the reasons I am pursuing this goal is because somebody else wants me to.”*) and (Introjected regulation: *“One of the reasons I am pursuing this goal is* because *I would feel ashamed, guilty, or anxious if I did not.”*) and two items of autonomous motivation (Identified regulation: *“One of the reasons I am pursuing this goal is because I really believe that it is an important goal to have.”*) and (Intrinsic regulation: *“One of the reasons I am pursuing this goal is because of the fun and enjoyment which the goal will provide*.”; Sheldon and Elliot, 1999). The self-concordance score was rated on a seven-point Likert scale (ranging from “Not at all true for me” to “Very true for me”). Due to the composite nature of the index, the standard reliability estimate of alpha is not applicable (Sheldon and Elliot, 1999).

##### 2.2.1.2. Goal self-efficacy

Goal self-efficacy refers to the person’s belief in his/her ability to achieve the goal. This four-item questionnaire provides a seven-point Likert scale for each response. Example item: *“I can handle the situations that come with achieving this goal.”* The self-efficacy score was rated on a seven-point Likert scale (ranging from “Not at all true for me” to “Very true for me”). Internal consistency of the four items was excellent: Cronbach’s α =0.805 (Rózsa et al., 2003).

##### 2.2.1.3. Positive and negative emotions

Six items referred to the emotional experiences during the goal implementation process. Three items were used for measuring negative (Example item: *“How often do you experience negative emotions on a daily basis: stress, worry, and anxiety about this goal?”*) and three items for positive emotions (Example item: *“How often do you experience positive emotions on a daily basis: joy and happiness about this goal?”*). Both subscales used a seven-point Likert scale (ranging from “Not at all true for me” to “Very true for me”). Internal consistency of the items for positive emotions was Cronbach’s α =0.806, and for the negative emotions, Cronbach’s α =0.890 (Martos et al., 2013).

#### 2.2.2. State–trait anxiety inventory

The Trait Anxiety Subscale was used to measure the general levels of anxiety. The trait anxiety score was calculated from 20 items, rated on a four-point Likert scale (ranging from “Almost Never” to “Almost Always”). Example items: *“I worry too much over something that really does not matter”* and *“I am content; I am a steady person.”* The scale had an internal consistency of 0.925 in our sample (Sipos, 1978; Spielberger, 1983).

### 2.3. Procedure

Our research was the pilot phase of a broader longitudinal study. The presented data were collected from April to May 2022 as a pilot study to test the self-concordance-based model’s reliability. Future phases of the research project, started in November 2022, will extend to three waves of longitudinal data collection. The paper-pencil questionnaire package, consisting of several other scales not discussed here, took approximately 30–40 min to complete. Only the Personal project analysis questionnaire is attached in Supplementary Material. The ethics approval was provided by the Regional Research Ethics Committee (RKEB) of the University of Szeged, Albert Szent-Györgyi Health Centre, under Nr. 14/2022-SZTE RKEB. The study was conducted following the Declaration of Helsinki.

### 2.4. Statistical analyses

JASP 0.14.6.0 was used for the statistical analyses of the data. Patients’ scores for the inventories were summarized using descriptive statistics, and Pearson’s correlation coefficients were used to quantify associations between variables (0.10 is small, 0.30 is moderate, and 0.50 is large; Cohen, 1988). Path analysis was used to examine the relationships between variables. To further examine the relationship between variables, standardized regression coefficients (β) were used to quantify the strength of association (0.10 is small, 0.30 is moderate, and 0.50 is large).

## 3. Results

### 3.1. Descriptive statistics of the sample

According to the type of IBD, 79 patients (56.02%) had Crohn’s disease (CD), 56 patients (39.71%) had a diagnosis of ulcerative colitis (UC), five patients (3.54%) reported having an unspecified type (UT) of IBD, and one patient (0.7%) did not know the type of the disease. Concerning the status of IBD, 95 patients’ disease was in remission (46.0%), and 44 patients’ disease was in the relapse phase (31.2%) at the time of the data collection, with two missing data (1.41%). The mean age of IBD subsamples for CD was 38.4 years (*SD* = 11.9), for UC, 39.70 years (*SD* = 13.3), and for UT, 54.0 years (*SD* = 18.7). Of CD patients, there were 46 female (58.22%), 31 male (39.24%) participants, and two persons with missing data (2.53%). UC patients involved 39 female (69.64%) and 14 male participants (25.0%), with three missing data (5.35%). From UT of IBD patients, there were three female and two male participants. Of the total sample, 101 patients (71.63%) reported having a health-related goal, and 40 patients (28.37%) reported not having a health-related goal. Of those with a health-related goal, 66 were female (68.75%) and 30 were male participants (31.25%), with five missing data. The IBD subsamples’ demographic information and other characteristics are summarized in Table 1.

**Table 1:** Demographic information and IBD characteristics.

Variable name	Type of IBD		
	UC	CD	UT
*N*	56	79	5
Age (*M, SD*)	38.65 (12.17)	39.00 (7.07)	37.35 (11.27)
*Gender*			
Male	8	19	2
Female	28	34	2
Missing	6		
*Level of education*			
Primary school	4	11	2
High school	18	16	1
College and higher	14	21	1
Missing	4		
*State of disease*			
Remission	19	38	1
Relapse	17	17	3
Missing	3		
*Psychological functioning (M, SD)*			
Trait Anxiety	44.64 (9.78)	40.98 (11.88)	49.25 (10.69)
State Anxiety	40. (9.61)	38.91 (11.80)	47.25 (14.25)
*Health-related goal*			
One or more	101 (%)		
None	40 (%)		

### 3.2. Correlations between goal characteristics and trait anxiety

We run a series of bivariate Pearson correlations for the study variables. According to the results, positive emotions, higher self-efficacy, and goal-self-concordance have a significant, weak to medium-strong negative association with the levels of trait anxiety (*p* was everywhere <0.001, *n* = 91–105): *r_PE_* = −0.37, *r_SC_* = −0.46, *r_SE_* = −0.36. Negative emotions had a significant, moderate positive association with trait anxiety (*r_NE_* = 0.43, *p* < 0.001). There was a significant positive association between SC and SE (*r* = 0.33, *p* < 0.001), and a significant negative association between PE and NE (*r* = −0.38, *p* < 0.001). Descriptive statistics and correlations are presented in Table 2.

**Table 2:** Correlations among goal self-concordance, goal self-efficacy, positive and negative emotions, and trait anxiety.

Variable	Mean	*SD*	1.	2.	3.	4.	5.
1. Positive emotions	4.27	1.32	–				
2. Negative emotions	3.27	1.77	−0.38**	–			
3. Self-concordance	2.81	2.22	0.31**	−0.34***	–		
4. Self-efficacy	4.89	1.21	0.48***	−0.26**	0.33***	–	
5. STAI-T	41.9	11.3	−0.37***	0.43***	−0.46***	−0.36***	–

(*n* = 91–105); **p < 0.01, ***p < 0.001.

### 3.3. Path analysis

#### 3.3.1. Model I

**Figure 2:**
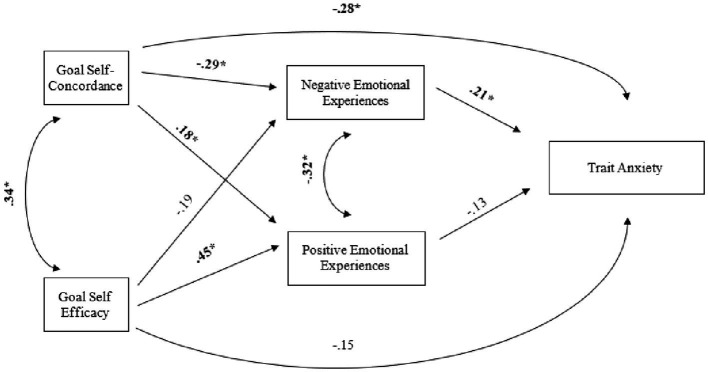
Path Diagram I. The diagram shows the standardized path coefficients. The sample size for the path model was *n* = 101. *p* for SE and NE was 0.06. *p < 0.05.

For path analysis, data were examined from individuals who reported having a health goal and completed all questions. Since our self-concordance-based model is saturated, the fit indices indicate a perfect fit to the data: *X*^2^ (0) = 0.00, *p* = 1.00, CFI = 1.00, TLI = 1.00, RMSE = 0.00, SRMR <0.001. Self-efficacy has a significant positive effect on positive emotions (*β* = 0.45 *p* < 0.001), and a significant negative effect on negative emotions (*β* = −0.19, *p* = 0.05). Self-concordance has a positive effect on positive emotions (*β* = 0.19, *p* = 0.06), and a significant negative effect on negative emotions (*β* = −0.27, *p* = 0.01). Positive emotions have no significant effect on trait anxiety (*β* = −0.13, *p* = 0.23), but negative emotions have a significant negative effect on trait anxiety (*β* = 0.21, *p* = 0.03). Self-concordance has a significant negative effect on trait anxiety (*β* = −0.28, *p* = 0.004). Self-efficacy has no significant effect on trait anxiety (*β* = −0.15, *p* = 0.16). Self-efficacy has a significant moderate positive association with self-concordance (*β* = 0.34, *p* = 0.002). Positive and negative emotions have a significant small negative association (*β* = −0.32, *p* = 0.002). Figure 2 shows defined paths.

#### 3.3.2. Model II

**Figure 3:**
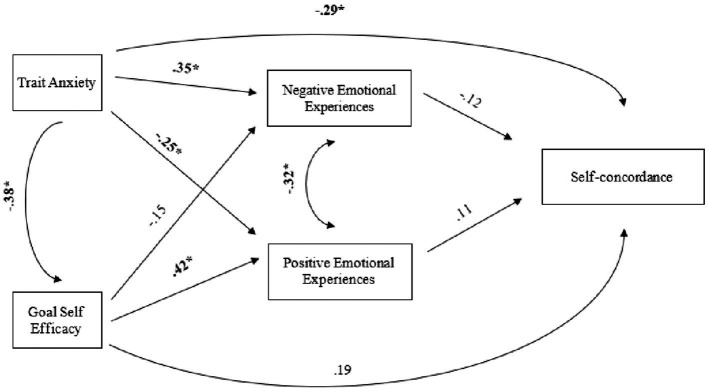
Path Diagram II. The diagram shows the standardized path coefficients. The sample size for the path model was *n* = 88.

In the absence of longitudinal data, we tested an alternative model for the pilot study to explore the effect of trait anxiety on self-efficacy and self-concordance. Since the alternative model is also saturated, the fit indices indicate a perfect fit to the data: *X*^2^ (0) = 0.00, *p* = 1.00, CFI = 1.00, TLI = 1.00, RMSE = 0.00, SRMR <0.001. Self-efficacy has a significant positive effect on positive emotions (*β* = 0.42*, *p* < 0.001), no significant effect on negative emotions (*β* = −0.15, *p* = 0.16), and no significant effect on self-concordance (*β* = 0.19, *p* = 0.08). Trait anxiety has a significant positive effect on negative emotions (*β* = 0.35, *p* < 0.001), a significant negative effect on positive emotions (*β* = −0.25, *p* = 0.01), a significant negative effect on self-concordance (*β* = −0.29, *p* = 0.005) and a significant negative effect on self-efficacy (*β* = −0.38, *p* < 0.001). Neither positive emotions (*β* = 0.11, *p* = 0.36) nor negative emotions significantly predict self-concordance (*β* = −0.12, *p* = 0.24). Positive and negative emotions have a significant small negative association (*β* = −0.31, *p* = 0.005). Figure 3 shows defined paths.

## 4. Discussion

Inflammatory bowel diseases (IBDs) are chronic conditions increasingly affecting a larger population worldwide (Goodhand et al., 2012). Patients with IBD have a higher prevalence of anxiety disorders compared to other chronic disorders, but the underlying factors require further investigation (Kovács and Kovács, 2007; Mikocka-Walus et al., 2016; Navabi et al., 2018). Effective self-management and adaptation to disease-specific lifestyle changes are crucial for maintaining and improving the health of IBD patients. This study examined a model based on goal self-concordance theory (Sheldon and Elliot, 1999) and found that more integrated goals are associated with increased positive affect and reduced negative affect, which, in turn, may contribute to better psychological functioning. By assessing patients’ autonomous motivation toward personal health goals, we provide evidence of how individuals with IBD can enhance their ability to manage their disease effectively.

The current results partially support our assumption that self-concordance represents an internal capacity leading to better self-management of IBD patients. Self-concordance significantly predicted lower levels of negative emotions and higher levels of positive emotions. Consistent with previous research, internally regulated goals are more likely to lead to activities that satisfy basic psychological needs and promote overall well-being (Ryan and Deci, 2000, 2017; Sheldon et al., 2004). The unidirectional relationship between positive and negative emotions and self-concordance reinforces the role of goal integration in enhancing positive emotions, as supported by the multivariate analysis.

A high level of goal self-concordance predicts reduced negative emotions, which, in turn, is associated with lower levels of trait anxiety. However, positive emotions do not significantly predict trait anxiety. This suggests a dynamic interaction between self-concordance and psychological functioning. Since goals are expressed through individual language, they reflect an individual’s ability to make accurate or inaccurate self-descriptions and reflect their state of self (Kuhl and Kazen, 1994; Sheldon and Elliot, 1999). Negative affect can impede conscious access to individuals’ extended personality system, resulting in inaccurate self-descriptions (Kuhl, 2000) and less self-concordant aspirations. According to our results, anxiety can hinder the ability to connect with their inner needs and formulate goals in a self-concordant manner. The results of this pilot study can guide future research on the role of anxiety in successful goal integration and the mechanisms by which self-concordance may improve self-management in patients with IBD.

We also hypothesized that self-efficacy would be associated with positive and negative emotions, and our results partly supported this hypothesis. In both models, self-efficacy was significantly related to positive emotions but did not show a significant association with negative emotions. Additionally, self-concordance significantly predicted trait anxiety, while trait anxiety also predicted self-efficacy. These distinct emotional patterns in self-concordant and self-effective goal striving support previous research indicating that although self-efficacy and self-concordance are linked, they represent different aspects of goal striving (Fuchs et al., 2016; Downes et al., 2017).

### 4.1. Limitations

Our study has several limitations that should be considered when interpreting the results. Firstly, the cross-sectional design of the data assessment prevents us from establishing causal relationships between variables. Future studies employing longitudinal designs would provide a clearer understanding of the causal effects. Secondly, the pilot study had a relatively small sample size, which may limit the generalizability of the findings. However, the effect sizes observed in our study can serve as a basis for determining sample sizes in larger studies involving IBD patients. Additionally, the small sample size could impact the precision of the estimated model parameters. Future studies with larger sample sizes would enhance the statistical power of the analyses (Wolf et al., 2013). Lastly, we could not assess goal attainment due to the cross-sectional design. Long-term assessments would be valuable in examining the importance of self-concordance for goal achievement.

### 4.2. Conclusion

The findings of our pilot study highlight the significance of health goal integration in more effective self-management and the psychological functioning of individuals with IBD. Health goals can be valuable tools for monitoring patients’ self-management processes, including successful lifestyle change and adherence. However, further research is needed to explore the complex role of goal integration in long-term well-being and the interplay between self-concordance, self-efficacy, and emotional experiences during disease management. By enhancing patients’ goal-related self-efficacy and self-concordance, clinicians can facilitate successful lifestyle changes and promote adherence in individuals living with IBD.

### 4.3. Plans and perspectives for future research

Based on the study, setting self-concordant goals is an internal capacity that can help patients with inflammatory bowel disease to maintain lifestyle changes and be more effective in disease management. This finding will serve as a basis for further research. Our research team is currently conducting a longitudinal study that will follow up with 300 IBD patients at 3 and 6 months to validate the proposed model and investigate causal relationships.

## Data availability statement

The raw data supporting the conclusions of this article will be made available by the authors, without undue reservation.

## Ethics statement

The studies involving human participants were reviewed and approved by the Regional Research Ethics Committee (RKEB) of the University of Szeged, Albert Szent-Györgyi Health Centre. The patients/participants provided their written informed consent to participate in this study.

## Author contributions

TMa, BH, AD, and SN led the data collection. BH and TMa done the study conceptualization, data cleaning, data analysis, and writing. AD, VS, BR, KO, MC, and TMo was done the review of the paper and interpretation of the results. All authors contributed to the article and approved the submitted version.

## Funding

The research was supported by research project no. K 138372, provided by the Ministry of Innovation and Technology of Hungary from the National Research, Development and Innovation Fund, and financed under the K_21 funding scheme.

## Conflict of interest

The authors declare that the research was conducted in the absence of any commercial or financial relationships that could be construed as a potential conflict of interest.

## Publisher’s note

All claims expressed in this article are solely those of the authors and do not necessarily represent those of their affiliated organizations, or those of the publisher, the editors and the reviewers. Any product that may be evaluated in this article, or claim that may be made by its manufacturer, is not guaranteed or endorsed by the publisher.
