# Supplementary material for: Improving disease management of patients with inflammatory bowel disease: the potential role of self-concordant health goals
Source: Front Psychol. 2023 Jul 3;14:1115160. doi: 10.3389/fpsyg.2023.1115160 (PMC10359902; doi:10.3389/fpsyg.2023.1115160)
Supplement: Supplementary file 1 [file Data_Sheet_1.docx]

Improving disease management of patients in inflammatory bowel disease: the potential role of self-concordant health goals

Supplementary Material

**Barbara Horvát*, Anett Dávid, Viola Sallay, Beatrix Rafael, Sanela Njers, Kata Orbán, Tamás Molnár, Márta Csabai, Tamás Martos**

*** Correspondence:** Corresponding Author: [horvat.barbara@med.u-szeged.hu](mailto:horvat.barbara@med.u-szeged.hu)

# Supplementary Questionnaires

## Personal Project Analysis (PPA, Little, 1993; Martos, 2009b)

MY PERSONAL GOALS for physical and mental health

In our everyday lives, we aim to achieve several large and small goals. We devote some time, attention, and effort to achieve these goals, and often (but not always) achieve them. In the following, we ask you to answer a few questions about these goals.

We will refer to these goals as your PERSONAL GOALS, e.g., a resolution, task, plan, undertaking, or project, which you have already achieved to some extent (i. e. not something you plan to start in the future).

First, we ask you to list some personal goals specific to your physical or mental health (e.g., lifestyle; weight; diet; exercise; using various health services and interventions; maintaining, and improving mental health). In other words, activities that you think impact your physical and mental health you think you would like to do in the next period (about 3-6 months).

As examples, here are some of the personal health goals that have been identified in our previous research given by participants in our previous surveys:

- I want to do physiotherapy properly.
- I want to keep my normal sleep time.
- I want a more balanced emotional life.
- I want to quit smoking.
- I want to strengthen my immune system.

Please indicate here if you currently have such a personal goal:

 I currently have a health-related personal goal.

 I do not currently have a health-related personal goal.

MY PERSONAL HEALTH-RELATED GOALS

Now we ask you to describe as many as possible, but at least one health-related personal goal! Of course, you can also mention very different goals from those above. Write down health-related goals that you consider to be relevant to you. The order does not matter.

At present, I want to achieve these PERSONAL GOALS related to my HEALTH:

……………………………………………………………………………………….

……………………………………………………………………………………….

……………………………………………………………………………………….

……………………………………………………………………………………….

Please select by underlining which of the goals you are currently working on. Choose the one you are most committed to in time, attention, and energy, not the one you are only generally or theoretically important. Even if you have set a single goal, mark it. We will ask you about your experience with this goal in the following.

ASSESSMENT OF A PERSONAL HEALTH-RELATED GOAL

You will be asked to describe again which of your health-related personal goals you have selected!

Please also write your chosen health-related goal here:

……………………………………………………………………………………………….

Below are various aspects which may be characteristic of this purpose. Please rate your personal goal from 1 to 7 for each criterion. The higher the value, the more you agree with the statement. The lower the value, the more you disagree with the statement.

Please mark which answers you feel are the most appropriate for your own experience! Please focus on the current aspect and do not consider any previous answers.

First, we ask you to think about what your personal experiences are related to this plan:

1. How often do you experience positive emotions on a daily basis: joy and happiness about this goal?

2. How often do you experience positive emotions on a daily basis: interest and curiosity about this goal?

3. How often do you experience positive emotions on a daily basis: contentment and peace of mind about this goal?

4. How often do you experience negative emotions on a daily basis: sadness and shame about this goal?

5. How often do you experience negative emotions on a daily basis: stress, worry, and anxiety about this goal?

6. How often do you experience negative emotions on a daily basis: anger and frustration about this goal?

We now list possible reasons why you might want to achieve this goal. Evaluate each statement in relation to your goal.

1 = not at all true for me 7 = very true for me

One of the reasons I am pursuing this goal is ...

1. … because somebody else wants me to.
2. … because I would feel ashamed, guilty, or anxious if I didn’t.
3. … because it is an important goal to have.
4. … because of the fun and enjoyment the goal provides.

We now ask you to think about how accurate the following statements are for you specifically in relation to this goal:

1. I can handle the situations that come with pursuing this goal.
2. It is easy for me to stick to my goal and achieve it.
3. With enough effort, I can solve almost any problem and find a solution.
4. I can handle unexpected situations while working towards my goal.
